# Evaluating a Hybrid Web-Based Training Program for Panic Disorder and Agoraphobia: Randomized Controlled Trial

**DOI:** 10.2196/20829

**Published:** 2021-03-04

**Authors:** Lara Ebenfeld, Dirk Lehr, David Daniel Ebert, Stefan Kleine Stegemann, Heleen Riper, Burkhardt Funk, Matthias Berking

**Affiliations:** 1 Leuphana University Lüneburg Germany; 2 Technical University of Munich Munich Germany; 3 GGZ ingeest Amsterdam Amsterdam Netherlands; 4 Vrije Universiteit Amsterdam Amsterdam Netherlands; 5 Amsterdam Public Health Research Institute Amsterdam Netherlands; 6 Friedrich-Alexander-University Nuremberg-Erlangen Nuremberg-Erlangen Germany

**Keywords:** panic disorder, agoraphobia, treatment, internet, mobile phone, randomized controlled trial

## Abstract

**Background:**

Previous studies provide evidence for the effectiveness of web-based interventions for panic disorder with and without agoraphobia. Smartphone-based technologies hold significant potential for further enhancing the accessibility and efficacy of such interventions.

**Objective:**

This randomized controlled trial aims to evaluate the efficacy of a guided, hybrid web-based training program based on cognitive behavioral therapy for adults with symptoms of panic disorder.

**Methods:**

Participants (N=92) with total scores in the Panic and Agoraphobia Scale ranging from 9 to 28 were recruited from the general population and allocated either to a hybrid intervention (GET.ON Panic) or to a wait-list control group. The primary outcome was the reduction in panic symptoms, as self-assessed using a web-based version of the Panic and Agoraphobia Scale.

**Results:**

Analysis of covariance-based intention-to-treat analyses revealed a significantly stronger decrease in panic symptoms posttreatment (*F*=9.77; *P*=.002; Cohen *d*=0.66; 95% CI 0.24-1.08) in the intervention group than in the wait-list control group. Comparisons between groups of the follow-up measures at 3 and 6 months yielded even stronger effects (3-month follow-up: *F*=17.40, *P*<.001, Cohen *d*=0.89, 95% CI 0.46-1.31; 6-month follow-up: *F*=14.63, *P*<.001, Cohen *d*=0.81, 95% CI 0.38-1.24).

**Conclusions:**

Hybrid web-based training programs may help reduce the symptoms of panic disorder and hence play an important role in improving health care for patients with this debilitating disorder.

**Trial Registration:**

German Clinical Trial Register DRKS00005223; https://tinyurl.com/f4zt5ran

**International Registered Report Identifier (IRRID):**

RR2-10.1186/1745-6215-15-427

## Introduction

With a 12-month prevalence of 1.8% among adults, panic disorder is one of the most common anxiety disorders [1,2]. Subthreshold cases, defined as significant panic symptoms that fail to meet full criteria, have been estimated to be just as prevalent [3,4] and have been shown to predict the development of full panic disorder as well as other mental disorders, such as generalized anxiety disorder or major depression [5]. Effective treatments for panic disorder and associated agoraphobic symptoms include pharmacotherapy and cognitive behavioral therapy (CBT) [6-9]. Unfortunately, many individuals still lack access to evidence-based treatments because of the limited availability of clinicians or fear of stigmatization [10-12].

Technology-based psychological interventions that use the internet provide low-threshold access to evidence-based mental health care. Recent outcome studies [13-15], meta-analyses, and reviews [16-24] provide ample evidence that internet-based interventions based on cognitive behavioral therapy principles (iCBT) are effective in treating panic disorder.

Owing to their ability to bridge distances between patients and therapists, good cost-efficacy and low-threshold iCBT have great potential to facilitate access to evidence-based interventions [18,25]. However, the current dominance of desktop-based iCBT in research and health services neglects the dramatic shift in user preferences toward the use of smartphones [26]. Moreover, smartphones accompany their users wherever they go, thereby providing an excellent opportunity for ecological momentary assessment of relevant health information [27-31]. Furthermore, smartphones allow the use of ecological momentary interventions to be delivered in the real world and real time, ideally at the very moment the intervention is needed [32]. Considering the rapid growth and potential of mobile technology, surprisingly little research has been conducted to clarify the benefits of using smartphones as stand-alone or add-on interventions [33]. Available data often come from studies criticized for poor-quality interventions [34], and many interventions currently available have not been evaluated at all [35-37].

The few currently available studies provide preliminary evidence for the efficacy of smartphone-based interventions for the symptoms of anxiety disorders. For example, in a meta-analysis on the efficacy of transdiagnostic eHealth interventions that integrated mobile technologies, Firth et al [38] showed that such interventions can significantly reduce overall anxiety (Hedge *g*=0.45). A recent study by Christoforou et al [39] evaluated the efficacy of an app for agoraphobic symptoms in comparison with a stress reduction app. Although there was a significant pre- to posttest effect for the interventions (Panic and Agoraphobia Scale [PAS] difference −5.97; 95% CI −8.49 to −3.44), no significant differences between the interventions were observed.

Despite these promising findings, it is important to acknowledge that mobile apps also have some disadvantages with regard to usability issues. For example, elaborate writing tasks, a typical component of iCBT interventions, are difficult to complete on a small screen with a smartphone touchpad. Moreover, cellphones are typically used for short time intervals and often while performing other tasks. This is problematic, as working toward health-promoting changes often requires more sustained and focused effort [40,41]. Therefore, it can be argued that hybrid interventions that combine the advantages of both desktop and mobile technology should be superior to exclusively desktop- or mobile-based approaches. In hybrid interventions, the mobile component can be used to monitor symptoms and cue exercises in the patient’s natural environment, whereas the desktop component provides text- and video-based psychoeducation and facilitates elaborate writing tasks.

Despite the obvious advantages of hybrid interventions, the literature on their efficacy is still scarce. In a transdiagnostic approach, Proudfoot et al [42] showed that the delivery of CBT using a combination of mobile app and desktop-based technology was effective in reducing symptoms of anxiety disorders (Cohen *d*=0.47) compared with a wait-list control (WLC) condition. Furthermore, in a study evaluating the combination of Acceptance and Commitment Therapy and a smartphone app for participants with panic disorder or social phobia, Ivanova et al [43] found no significant effect on panic symptom severity reduction. At this point, no study has been published on the efficacy of hybrid iCBT interventions for panic disorder. To fill this gap in the literature, this study aims to evaluate the efficacy of a newly developed hybrid iCBT training program for individuals with symptoms of panic disorder. Owing to the legal restrictions on remote treatment (*Fernbehandlungsgesetz*) [44], we use the term *online training program* for the intervention format instead of the term *online therapy*, which is more commonly used in the literature.

## Methods

### Study Design

To evaluate the efficacy of a hybrid web-based training program for panic disorder (with and without agoraphobia), we conducted a prospective, two-arm randomized trial, in which 92 participants with significant symptoms of panic disorder were randomly allocated either to the GET.ON Panic intervention group (IG) who received the training program immediately or to the WLC group who received the training program 6 months after randomization. For randomization, we used the automated computer program DatInf RandList version 1.2 (DatInf GmbH). The allocation was stratified for clinical or subclinical symptomatology as well as the presence or absence of agoraphobia in the order of incoming informed consent. To include equal numbers of participants in each group, we used block randomization (n=2 per block). The staff conducting the diagnostic interviews and observer ratings were blinded to the participants’ randomization statuses. The participants could participate in the training program with a pseudonym of their own choice. Ethical approval for this study was obtained from the Ethical Committee of the University of Marburg (registration number: 2013-23 K). The study was preregistered with the German Clinical Trial Register (registration number: DRKS00005223). The study protocol was submitted for publication before randomization [45].

### Participants and Recruitment

The study participants were recruited from the general population between August 2013 and October 2014. Announcements in newspapers, support groups, and social media, such as Facebook, guided interested individuals to the web-based health center website of our research group, where they could apply on the web to participate in the study. Applicants were asked to complete a web-based questionnaire assessing the following inclusion criteria: (1) experiencing mild-to-moderate panic symptoms as assessed by the PAS (score range: 9-28) [46,47], (2) being aged ≥18 years, (3) having panic as the primary concern for seeking help, (4) having internet and smartphone access with minimum system requirements of iPhone (TM) 3GS (Apple Inc; iOS 6 and iOS 7) or a comparable Android device (Android 2.3 or newer), and (5) providing their informed consent.

The exclusion criteria were as follows: (1) receiving current psychological help for anxiety problems or being on a wait-list for psychotherapy; (2) having physical health problems that were assessed via a self-report that prevents participants from engaging in self-exposure, as recommended by the German guideline for treating people with panic disorder and agoraphobia [48]; (3) currently having posttraumatic stress disorder or psychotic or dissociative disorders assessed via self-report and clinical interview; and (4) having current suicidality, as assessed by a score above 1 on item 9 of the Beck Depression Inventory II (BDI-II) [49,50] and question A9 of the Structured Clinical Interview for Diagnostic and Statistical Manual of Mental Disorders, Fourth Edition Axis I Disorders (SCID-I) [51]. In the event that potential participants were excluded because of suicidal ideation or intention, they were given information about further help according to an established suicide protocol. All excluded participants were contacted via email and provided with information regarding where they could obtain appropriate help.

### Treatment

Participants in the treatment condition received the GET.ON Panic treatment, which is a hybrid (ie, desktop-based and smartphone-based), iCBT-based self-help intervention for treating symptoms of panic disorder [45]. Participants were advised to log on to the training platform, which was provided by the technical partner Minddistrict GmbH on a weekly basis and consecutively work through the following sessions: (1) psychoeducation, (2) interoceptive exposure, (3) in vivo exposure, (4) cognitive restructuring—introduction, (5) cognitive restructuring—extension, and (6) relapse prevention. In addition, participants were instructed on the complementary use of the GET.ON Panic app [52]. The app supported participants in (1) completing their homework assignments (eg, keeping an anxiety diary); (2) planning, conducting, and evaluating interoceptive and in vivo exposure tasks; and (3) performing relaxation exercises (Table 1).

After every session, participants received written feedback from a trained coach based on a coaching manual developed by members of our research group to ensure a standardized procedure of coaching (the manual is available on request). The guidance focused on increasing motivation and adherence throughout the training progress, rather than providing individual therapeutic advice. The average feedback took about 20-30 min. Coaches also sent reminders via a secure messaging system within the training platform if participants did not log on for 1 week. All coaches had a degree in psychology and were supervised by a licensed clinical psychologist.

**Table 1:** Overview of sessions.

Week	Content and homework	
	Browser	Mobile
1	Psychoeducation: Information about panic Defining goals of training Setting up a reward list	Daily diary Registration of current panic event (event-based) Daily summary of panic, avoidance, and mood
2	Interoceptive exposure: Bodily symptoms during panic Avoidance Safety behaviors	Respiratory interoceptive exposure exercises Daily diary
3	In vivo exposure: Defining an anxiety hierarchy	In vivo exposures Dizziness interoceptive exercises Daily diary
4	Cognitive restructuring I: Negative automatic thoughts Defining anxiety project (training schedule for exposures)	In vivo exposures Further interoceptive exposure exercises Daily diary
5	Cognitive restructuring II: Reality testing of automatic negative thoughts	In vivo exposures Further interoceptive exposure exercises Daily diary
6	Relapse prevention: Early warning signs Critical life events Evaluation of training and aims	Breathing and muscle relaxation exercises

### Outcome Measures

#### Panic Symptom Severity and Self-Rating

The primary outcome was the severity of panic and agoraphobia symptoms, as self-assessed using the PAS (German version: Panik- und Agoraphobieskala) [46,47,53,54]. This scale consists of 13 items separated into the subscales of panic attacks, agoraphobic avoidance, anticipatory anxiety, daily life limitations, and health concerns. For each item, participants rated the frequency of panic symptoms during the past week on a 5-point scale. Thus, the total score ranges from 0 to 52, with scores ranging from 0 to 8 indicating *no clinically relevant symptoms*, scores ranging between 9 and 28 indicating *moderate symptoms*, and a score of 29 and above indicating a *severe level of symptoms*. Previous studies provide evidence for the efficacy of the measures, for example, Cronbach *α*=.86 [47] or *α*=.70 to .94 [55]. In this study, Cronbach *α* for the total score was .89.

#### Observer-Rated Anxiety Symptoms

The Hamilton Anxiety Scale (HAM-A) [56-58] was used as a complement for the self-administered anxiety scales. The scale contains 14 items, with a total score ranging from 0 to 30. Previous studies showed excellent interrater and test-retest reliabilities of intraclass correlation coefficients of 0.89-0.99 [57]. To examine interrater reliability in this trial, we audiotaped all the observer ratings. Around one-tenth (equivalent to 28 interviews) of these audio files were rated by experienced, blinded second raters. The interrater reliability was excellent, with an intraclass correlation coefficient of 0.99.

#### Agoraphobic Cognitions

The Agoraphobic Cognitions Questionnaire (ACQ) is a 14-item self-report questionnaire that measures agoraphobic cognitions. The total sum score of the ACQ ranged from 1 to 5. The ACQ has an internal reliability of Cronbach *α* of .80 [59-61]. In this trial, we found a Cronbach *α* of .84.

#### Body Sensations

Bodily sensations were measured using the Body Sensations Questionnaire (BSQ), a self-rating questionnaire with the total score ranging from 1 to 5 points. It has good internal reliability of Cronbach *α* of .87 [59-61]. In this trial, Cronbach *α* was .86.

#### Agoraphobic Avoidance

The Mobility Inventory (MI) is a questionnaire that measures agoraphobic avoidance. Participants were asked to rate common agoraphobic situations with regard to their avoidance. Each item is rated twice: once for dealing with the situation alone and once when accompanied. The total score ranged from 1 to 5 points. The internal consistencies reported in previous studies were very good, with Cronbach *α* of .91 (accompanied by significant others) and .94 (alone) [59,61,62]. In this study, Cronbach *α* values of the MI were .93 (accompanied) and .95 (alone).

#### Depressive Symptoms

We used the German adaption (ADS) of the Center for Epidemiologic Studies Depression Scale (CES-D) to assess depressive symptom severity. The CES-D measures 20 symptoms of depression in the previous week. The total score ranges from 0 to 60. Internal consistency has been shown to be good (Cronbach *α*=.89) [63,64]. In this study, Cronbach *α* was .87.

#### Diagnostic Status

The presence of panic disorder, any other anxiety disorder, or a current depressive episode was assessed using a telephone version of the SCID-I at the 6-month follow-up (6M-FU) assessment covering the period of the last 3 months by trained interviewers. Previous studies have shown excellent test-retest reliability between the 2 different formats, the telephone version and the face-to-face (f2f) version of the diagnostic interview (Cohen =0.84) [65-67]. To determine the interrater reliability of the diagnostic interviews, we used the statistics. In a previous study, moderate interrater reliability (Cohen =0.67) was found [68]. In this trial, all interviews were audiotaped, with 11.1% (18/162) of the interviews rated by an experienced, blinded second rater. Agreement between the 2 raters was moderate, with a Cohen of 0.51.

#### Quality of Life

Quality of life was measured using the 12-item Short-Form Health Survey (SF-12), which assesses 8 health domains: physical functioning, role limitations, pain, general health perception, vitality, mental health, emotional role, and social functioning. The SF-12 provides 2 summary scores for physical and mental health [69,70]. In this trial, Cronbach *α* was .79.

#### User Satisfaction

We assessed user satisfaction with the Client Satisfaction Questionnaire adapted to internet-based interventions (CSQ-I) [71], which is based on the German version of the Client Satisfaction Questionnaire [72,73]. Statements such as “I would recommend this training to a friend, if he or she was in need of similar help” are rated on a 4-point Likert scale (ranging from 1=*does not apply to me* to 4=*does totally apply to me*). The questionnaire contained 8 items, with a total score ranging from 8 to 32. The psychometric properties were excellent with a McDonald ω of 0.93 and ω of 0.95 [71]. In this trial, McDonald ω was 0.97.

### App Usage

The mobile app contains a diary for recording and monitoring panic-related symptoms, such as panic events, degree of avoidance behavior, general anxiety, and mood level on a visual analog scale (0-10). Furthermore, the app recorded the type and number of exposure exercises performed by the participant. In addition, we used the System Usability Scale (SUS) at postassessment (T2) to assess the usability of the GET.ON Panic app [74,75]. The sum score ranges from 0 to 100, with a higher score indicating better usability.

### Assessment Schedule

Participants completed a sociodemographic questionnaire, the PAS and the Suicidality item of BDI-II at screening (T0); at baseline (T1), we assessed the PAS, the SCID-I, the HAM-A, the ACQ, the BSQ, the MI, the CES-D, and the SF-12; at postassessment (T2), we assessed the PAS, the HAM-A, the ACQ, the BSQ, the MI, the CES-D, the SF-12, the CSQ-I (only IG), and the SUS (only IG); at 3-month follow-up (3M-FU; T3), we assessed the PAS, the ACQ, the BSQ, the MI, the CES-D, and the SF-12; and at 6M-FU (T4), we assessed the PAS, the SCID-I, the HAM-A, the ACQ, the BSQ, the MI, the CES-D, and the SF-12. Diary data were continuously measured during the training period and beyond (Table 2).

**Table 2:** Assessment schedule.

Assessments	Screening	T1	T2	T3	T4
Sociodemographic questionnaire	X^a^	—^b^	—	—	—
Suicidality (Item 9; BDI-II^c^)	X	—	—	—	—
Diagnosis (SCID-I^d^, sections for anxiety disorders and current depressive episode)	—	X	—	—	X
Panic and agoraphobia severity, self-rating (PAS^e^)	X	X	X	X	X
Panic and agoraphobia severity, observer-rating (HAM-A^f^)	—	—	X	—	X
Agoraphobic cognitions (ACQ^g^)	—	X	X	X	X
Body sensations (BSQ^h^)	—	X	X	X	X
Agoraphobic avoidance (Mobility Inventory)	—	X	X	X	X
Depressive symptoms (CES-D^i^)	—	X	X	X	X
Quality of life (SF-12^j^)	—	X	X	X	X
User satisfaction (CSQ-I^k^)	—	—	(X)^l^	—	—
Usability of smartphone app (SUS^m^)	—	—	(X)	—	—

^a^Measured.

^b^Not measured.

^c^BDI- II: Beck Depression Inventory II.

^d^SCID-I: Structured Clinical Interview for Diagnostic and Statistical Manual of Mental Disorders, Fourth Edition Axis I Disorders.

^e^PAS: Panic and Agoraphobia Scale.

^f^HAM-A: Hamilton Anxiety Scale.

^g^ACQ: Agoraphobic Cognitions Questionnaire.

^h^BSQ: Body Sensations Questionnaire.

^i^CES-D: Center for Epidemiologic Studies Depression Scale.

^j^SF-12: 12-Item Short-Form Health Survey.

^k^CSQ-I: Client Satisfaction Questionnaire adapted to internet-based interventions.

^l^Only available for intervention group.

^m^SUS: System Usability Scale.

### Statistical Analyses

To assess treatment efficacy, the GET.ON Panic group was compared with the WLC group on all outcome measures (T2, T3, and T4) using univariate analyses of covariance (ANCOVAs) with the baseline scores as covariates. On the basis of a previous meta-analysis [21], we powered the study to detect an effect size of Cohen *d*=0.6 (1− of 80%; =.05) with intention-to-treat (ITT) at T2 as our primary level of analysis. Accordingly, a sample size of 90 was required. Cohen *d* [76] was used to measure effect size. To account for covariance, we calculated Cohen *d* over the partial eta squared (*η^2^*). To assess a clinically reliable change of panic severity (response) on an individual level, we calculated the Reliable Change Index (RCI) as proposed by Jacobson and Truax [77], coded participants as responders or deteriorators if their score on the PAS differed by 10.68 points on the PAS, and performed a Pearson chi-square test to compare the reliable change of the GET.ON Panic group with the WLC. Corresponding to the RCI, we calculated numbers needed to treat (NNT) score indicating how many participants must take part in order for GET.ON Panic to achieve one clinically relevant improvement. To assess remission rates, we calculated the percentage of people who had a diagnostic status of panic disorder according to the SCID-I interview at baseline (T0) and at the 6M-FU (T4) and performed a Pearson chi-square test to compare the diagnostic status of the GET.ON Panic group with the WLC covering a period of the last 3 months.

Missing data at postassessment, 3M-FU assessment, and 6M-FU assessment were performed using a Markov Chain Monte Carlo multivariate imputation algorithm (SPSS 23) with hundred estimations per missing value and all available data on outcomes, age, and gender as predictors [78].

## Results

### Enrollment

**Figure 1:**
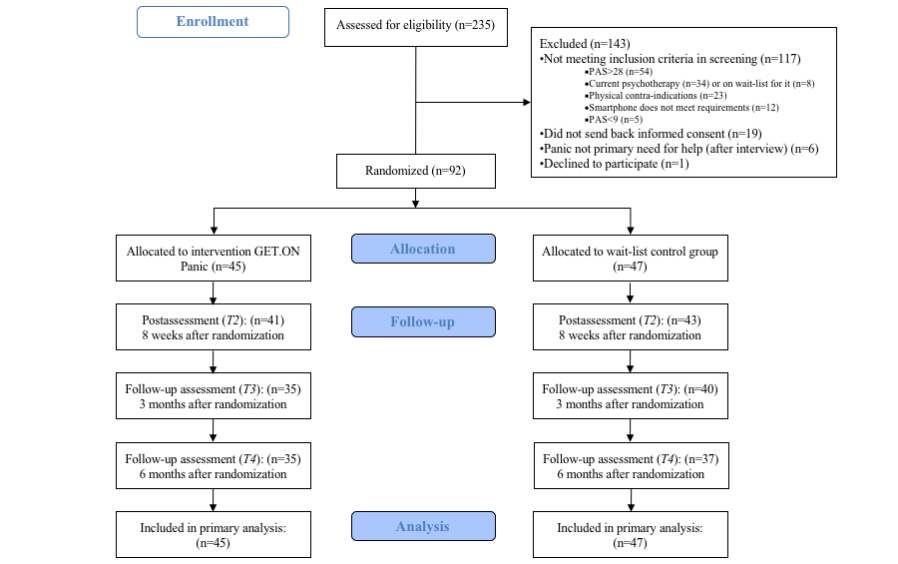
Study flow. PAS: Panic and Agoraphobia Scale.

Over a period of 14 months, a total of 235 individuals completed the screening questionnaire. Of these, 117 did not meet the inclusion criteria or matched one or more exclusion criteria (Figure 1). Severe panic symptom severity (n=54), current psychotherapy (n=34), or physical contraindications (n=23) were the most frequent reasons for exclusion. The remaining 118 candidates were eligible to participate in the clinical interviews. Of those, 19 did not provide informed consent. After this interview, another 6 candidates were excluded because they did not have panic symptoms as their primary reason for seeking help. All excluded individuals were provided with information about applicable health care system services. The remaining 92 participants were randomly assigned to the hybrid web-based training program GET.ON Panic or the WLC condition.

### Baseline Characteristics

Most participants were female (51/92, 55%), White (76/92, 83%), on average aged 38 years (SD 10.42), highly educated (60/92, 65%), married or in a relationship (82/92, 89%), and currently working (51/92, 55%). On the basis of the SCID interview, the most common diagnosis was panic disorder with agoraphobia (78/92, 83%). A significant number of patients (12/92, 13%) met the criteria for panic disorder without agoraphobia. Of all participants, 26% (24/92) met the criteria for at least one additional anxiety disorder, in addition to panic disorder. A small percentage (2/92, 2%) had a current major depressive episode as a comorbid condition. Most participants (58/92, 63%) reported that they had previously undergone psychotherapeutic treatment (Multimedia Appendix 1).

### Study Dropout and Compliance in Treatment

Baseline data were available for all the participants. The attrition rate was 9% (8/92) at postassessment (4/45 in the IG and 4/47 in the WLC), 18% (17/92) at the 3M-FU (10/45 in the IG and 7/47 in the WLC), and 22% (20/92) at the 6M-FU (10/45 in the IG and 10/47 in the WLC; Figure 1).

On average, the number of completed sessions in the GET.ON Panic group was 5.11 (SD 1.67). All 6 sessions were completed by 73% (33/45) of the participants, 4% (2/45) of the participants completed only session 1, 11% (5/45) of the participants dropped out after session 2, 7% (3/45) of the participants were lost after session 3, 2% (1/45) of the participants stopped the training after completing session 4, and 2% (1/45) of the participants after session 5. In total, 27% (12/45) of the participants did not complete the training. The reasons for intervention dropout were mentioned for 33% (4/12) of them: lack of time, lack of motivation, lack of personal contact with the eCoach, or surgery that interfered with completing the intervention. The resting 67% (8/12) of the participants were also study dropouts, and no reasons for stopping the intervention were known because they did not complete the postassessment.

### Severity of Panic Symptoms

Preliminary analyses indicated that all necessary conditions for the intended statistical analyses were met. There was a greater decrease in self-reported panic disorder symptom severity in the intervention condition than in the WLC condition (Multimedia Appendices 2 and 3). With regard to the primary outcome, participants in the GET.ON Panic condition reported significantly lower (baseline-controlled) panic symptom severity at posttreatment than the WLC group (*F*=9.77; *P*=.002; partial *η^2^*=0.10; Cohen *d*=0.66; 95% CI 0.24-1.08). This effect became even stronger (*F*=17.40; *P*<.001; partial *η^2^*=0.16; Cohen *d*=0.89; 95% CI 0.46-1.31) at the 3M-FU and remained significant (*F*=14.63; *P*<.001; partial *η^2^*=0.14; Cohen *d*=0.81; 95% CI 0.38-1.24) at the long-term 6M-FU. The effect sizes ranged from medium to large.

With regard to observer-based ratings, ANCOVA showed a significant difference in anxiety symptoms between groups as measured by the HAM-A at postmeasurement (*F*=3.97; *P*=.049; partial *η^2^*=0.04; Cohen *d*=0.42; 95% CI 0.01-0.84) and at the 6M-FU (*F*=4.86; *P*=.03; partial *η^2^*=0.05; Cohen *d*=0.47; 95% CI 0.05-0.88) with small-to-medium effect sizes. Further analyses indicated that the findings did not significantly change when the analyses were based on the study completer instead of the ITT sample.

With regard to response, the reliable clinical changes were not significant at postmeasurement (*χ*^2^_2_ [n=92]=2.5; *P*=.28; improvement: GET.ON Panic: 12/45, 27% and WLC: 7/47, 15%; deterioration: GET.ON Panic: 2/45, 4% and WLC: 1/47, 2%) or at the 3M-FU (χ^2^_2_ [*n*=92]=5.3; *P*=.07; improvement: GET.ON Panic: 14/45, 31% and WLC: 6/47, 13%; deterioration: GET.ON Panic: 0/45, 0% and WLC: 1/47, 2%). However, the GET.ON Panic group was superior to the WLC in terms of the percentage of participants attaining reliable clinical change (RCI=±10.68) in panic symptom severity at the 6M-FU (χ^2^_2_ [n=92]=6.0; *P*=.049; improvement: GET.ON Panic: 22/45, 49% and WLC: 12/47, 26%; deterioration: GET.ON Panic: 0/45, 0% and WLC: 1/47, 2%). These reliable clinical changes correspond to NNT from baseline to posttreatment of 8.49 (95% CI 3.54 to >10^6^), at the 3M-FU of NNT=5.45 (95% CI 2.87-55.78), and at the 6M-FU of NNT=4.28 (95% CI 2.35-24.07). Regarding the long-term effect, these results indicate that 4 individuals had to participate in the GET.ON Panic training program to result in one additional individual having reliable clinical improvement in panic symptom severity.

With regard to remission rates, at baseline, nearly all participants (90/92, 98%) fulfilled the diagnostic criteria for panic disorder. At the 6M-FU, 76% of the participants (70/92, 76% GET.ON Panic group: 33/45, 73%; WLC: 37/47, 79%) agreed to the telephone-administered diagnostic interview. In total, 21% (15/70) of the participants were free of a diagnosis. There was a greater reduction in diagnoses in the GET.ON Panic group (11/33, 33%) than in the WLC group (4/37, 11%; χ^2^_1_[n=70]=5.3; *P*=.02).

### Additional Anxiety Measures

Comparing the GET.ON Panic with the WLC group on further self-rated anxiety measurements, we found stronger between-group effect sizes for agoraphobic cognitions (partial *η^2^*=0.06; Cohen *d*=0.51; 95% CI 0.05-0.93) and bodily sensations (partial *η^2^*=0.05; Cohen *d*=0.46; 95% CI 0.05-0.88) in the GET.ON Panic group than in the WLC group at posttreatment. With regard to follow-up measurements, these effects remained stable for both agoraphobic cognitions (partial *η^2^*=0.07; Cohen *d*=0.55; 95% CI 0.14-0.97 after 3 months and partial *η^2^*=0.05; Cohen *d*=0.46; 95% CI 0.04-0.87 after 6 months) and bodily sensations (partial *η^2^*=0.14; Cohen *d*=0.79; 95% CI 0.37-1.22 after 3 months and partial *η^2^*=0.09; Cohen *d*=0.66; 95% CI 0.22-1.06 after 6 months). A difference in agoraphobic avoidance between the groups could only be found when participants had to manage difficult situations when they were alone with small effect sizes (partial *η^2^*=0.05; Cohen *d*=0.45; 95% CI 0.04-0.86) at posttreatment and a medium effect size (partial *η^2^*=0.11; Cohen *d*=0.70; 95% CI 0.27-1.12) at the 6M-FU. ANCOVA did not reveal a significant difference between the groups regarding agoraphobic avoidance when participants had to manage difficult situations when they were in companionship with other people (Multimedia Appendices 2 and 3).

### Additional Measures

At the postmeasurement as well as at the 3M-FU, the GET.ON Panic group showed no significant reduction in depressive symptoms compared with the WLC group (Multimedia Appendices 2 and 3). However, at the 6M-FU, the depressive symptoms of the GET.ON Panic group decreased significantly with a small effect size compared with the WLC group (partial *η^2^*=0.06; Cohen *d*=0.49; 95% CI 0.07-0.90). The results on the quality of life scales with regard to mental health showed no reduction at postmeasurement or at the 3M-FU but a medium reduction after 6 months (partial *η^2^*=0.11; Cohen *d*=0.70; 95% CI 0.28-1.12). Furthermore, no differences in symptoms regarding physical aspects of quality of life were found.

### App Usage and User Satisfaction

The participants of the training group (n=45) used the mobile diary on average 25.02 times (SD 19.48; range 0-56) during the 8-week training period on average (0.45 diary entries per day per participant). The repeated analysis of variance did not reveal any changes in the diary scores over a period of 8 weeks. Furthermore, they were not related to the primary outcome. The participants performed an average of 149.80 (SD 279.34; range 0-1702) interoceptive exposure exercises and 6.63 in vivo exercises (SD 17.74; range 0-113). The mean SUS score was 71.16 (SD 18.97) at posttreatment, which indicates good usability of the GET.ON Panic app. Overall, user satisfaction with the hybrid training program was high (mean 28.10, SD 5.09). For example, 91% of the participants indicated that they would recommend the training program to a friend in need.

## Discussion

### Principal Findings

This study aims to evaluate the efficacy of GET.ON Panic, a guided, mobile- and web-based CBT training program for adults with significant panic disorder symptoms. The results show that individuals treated with GET.ON Panic experienced a significantly greater reduction in panic disorder symptom severity than did participants in a WLC condition with between-group effect sizes of Cohen *d*. The findings also show that the effects were not only stable over time but even increased after the treatment was completed (Cohen *d*/NNT=0.66/8.49 at posttreatment vs Cohen *d*/NNT=0.89/5.45 after 3 months and Cohen *d*/NNT=0.81/4.28 after 6 months).

### Comparison With Prior Work

As such, they fall well into the range of reported effect sizes in meta-analyses for internet-based interventions for panic disorder (eg, Hedge *g*=0.83 [18]; *g*=1.31 [19]; Hedge *g*=0.83 [21]; Cohen *d*=0.96 [24]). The findings also showed that one of 3 participants in the IG had attained complete remission of panic disorder at the last assessment point, whereas this was only the case in one of 10 participants in the control group. With regard to secondary outcomes, it is of note that the 6M-FU effects on depressive symptoms are larger than the average of effects reported for psychological treatment for depression (Cohen *d*=0.49 vs Hedge *g*=0.35 [79]). Finally, in this study, adherence rates and user satisfaction were slightly higher than those reported in previous studies (adherence: 73% vs 66%; satisfaction: 91% vs 86% [19]).

A potential step-up could be the use of an intervention that integrates hybrid web-based training program into f2f CBT [80]. In such blended interventions, therapists might fully exploit the potential of using the ecological momentary assessment data provided by the smartphone as well as the potential of ecological momentary interventions derived from individual case formulations and carried into the patient’s life with the help of their mobile devices [81]. The adherence and usability rates in this study appear to be superior to what is currently reported for desktop-based iCBT interventions. This suggests that the integration of mobile components into iCBT should be a focus of future studies. The rapidly shifting use of mobile- instead of desktop-based devices underlines this [26,82].

The finding that depressive symptom severity was significantly reduced in the IG is important, as many individuals with panic disorder also have other mental health problems such as depression [83]. As cooccurring disorders may mutually help maintain each other [5,84], it is important that comorbid conditions are treated along with the primary disorder. The positive effects of the hybrid intervention evaluated in this study on depression are consistent with the findings from CBT that successfully treat panic disorder, which also result in a reduction of depressive symptoms [85,86].

### Strengths and Limitations

To our knowledge, this is the first study to examine the efficacy of iCBT training program that makes use of mobile components in a target group of people with mild-to-moderate panic and agoraphobia symptoms. One of the main strengths of this study is its solid study design, which tests a newly developed training program within a randomized controlled trial against a WLC. In addition to self-rating outcomes, we conducted clinical interviews with regard to symptom severity and changes in diagnostic status over a period of 6 months and an observer-rated anxiety outcome to validate the outcomes based on self-ratings. Furthermore, this study has high ecological validity, as participants used their own smartphones. They were supposed to interact with their smartphones as they would normally do. This may lead to a higher acceptance of and satisfaction with the GET.ON Panic training program and foster the integration of psychological interventions into the daily lives of individuals. The overall low dropout rates in this study support this assumption.

This study has several limitations that need to be considered. First, the study results cannot be generalized to all individuals with panic disorder symptoms. Participants who took part in this trial actively participated and underwent an extended eligibility procedure before the study. Many interested individuals were excluded based on the criteria defined in the study protocol. Thus, we assume that the current participants represent a more intrinsically motivated study sample and, in addition, have a higher affinity for the internet than the average individuals with panic disorder. Therefore, the external validity of this study might be limited. Second, for future treatment development, it would have been of interest to compare the hybrid intervention with both an exclusively desktop-based and an exclusively mobile-based intervention for panic disorder. However, as such a design was beyond what we could realize in this study, it would need to be used in subsequent studies. Such studies should also compare the efficacy (and cost-effectiveness) of desktop-, mobile-based, hybrid, and blended interventions with f2f therapy for panic disorder. Third, we cannot draw any conclusions on the efficacy beyond the 6M-FU assessment. Thus, future studies should evaluate the long-term effects of hybrid iCBT interventions for panic disorder.

### Conclusions

The results of this study suggest that a significant number of individuals with symptoms of panic disorder can be helped with an intervention that is comparatively easy to disseminate and that can be used anonymously, which arguably lowers an important barrier to service utilization [87]. However, the results also show that about two-thirds of the participants had not completely recovered after the intervention. Thus, interventions such as GET.ON Panic might best be used in a step-by-step care framework in which patients failing to attain recovery through an internet-based intervention subsequently receive more intense (and costly) interventions [88].

## Appendix

Multimedia Appendix 1 Characteristics of the study participants (N=92) allocated to online training GET.ON intervention group (n=45) and wait-list control group (WLC) (n=47) at baseline.

Multimedia Appendix 2 Mean and SD values of all outcome variables at baseline, posttreatment, and at 3-month and 6-month follow-ups (intention-to-treat, N=92).

Multimedia Appendix 3 Differences between groups at T2, T3, and T4 (intention-to-treat, N=92).

Multimedia Appendix 4 CONSORT-eHEALTH checklist (V 1.6.1).

## Abbreviations

3M-FU: 3-month follow-up
6M-FU: 6-month follow-up
ACQ: Agoraphobic Cognitions Questionnaire
ANCOVA: analysis of covariance
BDI-II: Beck Depression Inventory II
BSQ: Body Sensations Questionnaire
CBT: cognitive behavioral therapy
CES-D: Center for Epidemiologic Studies Depression Scale
CSQ-I: Client Satisfaction Questionnaire adapted to internet-based interventions
f2f: face-to-face
HAM-A: Hamilton Anxiety Scale
iCBT: internet-based interventions based on cognitive behavioral therapy principles
IG: intervention group
ITT: intention-to-treat
MI: mobility inventory
NNT: numbers needed to treat
PAS: Panic and Agoraphobia Scale
RCI: Reliable Change Index
SCID-I: Structured Clinical Interview for Diagnostic and Statistical Manual of Mental Disorders, Fourth Edition Axis I Disorders
SF-12: 12-Item Short-Form Health Survey
SUS: System Usability Scale
WLC: wait-list control
