# Supplementary material for: Evaluating a Hybrid Web-Based Training Program for Panic Disorder and Agoraphobia: Randomized Controlled Trial
Source: J Med Internet Res. 2021 Mar 4;23(3):e20829. doi: 10.2196/20829 (PMC7974752; doi:10.2196/20829)
Supplement: Multimedia Appendix 1 [file jmir_v23i3e20829_app1.docx]

Table 3. Characteristics of Study Sample (N=92) Allocated to Online Training GET.ON Panic (n=45) and Wait-list Control Group (WLC) (n=47) at Baseline.

|  | All | GET.ON Panic | WLC |
| --- | --- | --- | --- |
| Sociodemographic characteristics |  |  |  |
| Age in years, M (SD) | 38.36 (10.42) | 39.33 (10.83) | 37.43 (10.03) |
| Gender, n (%) female | 51 (55.4) | 27 (60.0) | 24 (51.1) |
| Ethnicity, n (%)  White  Asian  NA | 76 (82.6)  1 (1.1)  15 (16.3) | 38 (84.4)  7 (15.6) | 38 (80.9)  1 (2.1)  8 (17.0) |
| Marital status, n (%)  Never married  Married or in a partnership  Divorced or widowed | 37 (40.2)  48 (52.2)  7 (7.6) | 16 (35.6)  27 (60.0)  2 (4.4) | 21 (44.7)  21 (44.7)  5 (10.6) |
| Educational level, n (%)  Low (primary)  Middle (secondary)  High (A-level or higher) | 7 (7.6)  25 (27.2)  60 (65.2) | 3 (6.7)  11 (24.4)  31 (68.9) | 4 (8.5)  14 (29.8)  29 (61.7) |
| Employment, n (%)  Full-time  Part-time  Nonworking  Unemployed  On sick leave | 52 (56.5)  30 (32.6)  6 (6.5)  1 (1.1)  3 (3.3) | 27 (60.0)  15 (33.3)  3 (6.7) | 25 (53.2)  15 (31.9)  3 (6.4)  1 (2.1)  3 (6.4) |
| Clinical characteristics |  |  |  |
| Severity of panic symptoms (PAS), M (SD) | 18.82 (6.03) | 18.18 (6.54) | 19.43 (5.49) |
| Primary diagnosis, n (%)  Panic Disorder with Agoraphobia  Panic Disorder without Agoraphobia  Subclinical symptoms of PD/A or PD | 78 (84.8)  12 (13)  2 (2.2) | 39 (86.7)  5 (11.1)  1 (2.2) | 39 (83)  7 (14.9)  1 (2.1) |
| Comorbidity (one or more), n (%)  Depression (MDE)  Social Anxiety Disorder  Specific Phobia  Obsessive Compulsive Disorder  Generalized Anxiety Disorder | 24 (26.1)  2 (2.2)  3 (3.3)  14 (15.2)  1 (1.1)  4 (4.3) | 13 (28.9)  2 (4.4)  9 (20)  2 (4.4) | 11 (23.4)  2 (4.3)  1 (2.1)  5 (10.6)  1 (2.1)  2 (4.3) |
| Former experience, n (%)  Psychotherapy  For anxiety symptoms  Mental training | 58 (63)  50 (54.3)  23 (25) | 29 (64.4)  23 (51.1)  14 (31.1) | 29 (61.7)  27 (57.4)  9 (19.1) |
| Use of anxiolytic medication, n (%) | 22 (23.9) | 12 (26.7) | 10 (21.3) |
