# Supplementary material for: Evaluating a Hybrid Web-Based Training Program for Panic Disorder and Agoraphobia: Randomized Controlled Trial
Source: J Med Internet Res. 2021 Mar 4;23(3):e20829. doi: 10.2196/20829 (PMC7974752; doi:10.2196/20829)
Supplement: Multimedia Appendix 2 [file jmir_v23i3e20829_app2.docx]

Table 4. Means and Standard Deviations of all outcome variables at baseline, post-treatment, at 3-month and 6-month follow-up (intention-to-treat, N=92).

| Outcome | T1 | | | | T2 | | | | T3 | | | | T4 | | | |
| --- | --- | --- | --- | --- | --- | --- | --- | --- | --- | --- | --- | --- | --- | --- | --- | --- |
|  | GET.ON Panic | | WLC | | GET.ON Panic | | WLC | | GET.ON Panic | | WLC | | GET.ON Panic | | WLC | |
|  | Mean | SD | Mean | SD | Mean | SD | Mean | SD | Mean | SD | Mean | SD | Mean | SD | Mean | SD |
| Panic and agoraphobia severity, self-rating (PAS) | 18.18 | 6.54 | 19.43 | 5.49 | 11.97 | 6.66 | 17.20 | 8.58 | 9.99 | 6.79 | 16.02 | 6.66 | 8.79 | 0.84 | 14.01 | 6.93 |
| Anxiety symptoms, observer-rating (HAM-A) | 15.67 | 8.58 | 16.09 | 7.08 | 12.82 | 6.48 | 15.43 | 7.88 | NA | NA | NA | NA | 10.06 | 6.92 | 12.96 | 7.41 |
| Agoraphobic cognitions (ACQ) | 1.88 | 0.42 | 1.99 | 0.53 | 1.66 | 0.43 | 1.93 | 0.66 | 1.61 | 0.53 | 1.94 | 0.59 | 1.63 | 0.50 | 1.89 | 0.53 |
| Body sensations (BSQ) | 2.44 | 0.61 | 2.47 | 0.51 | 2.16 | 0.60 | 2.40 | 0.66 | 1.94 | 0.55 | 2.34 | 0.68 | 1.92 | 0.41 | 2.23 | 0.66 |
| Agoraphobic avoidance (MI), accompanied | 1.53 | 0.59 | 1.64 | 0.61 | 1.47 | 0.53 | 1.58 | 0.61 | 1.43 | 0.56 | 1.59 | 0.61 | 1.37 | 0.47 | 1.54 | 0.59 |
| Agoraphobic avoidance (MI), alone | 1.92 | 0.71 | 2.06 | 0.85 | 1.75 | 0.68 | 2.03 | 0.90 | 1.70 | 0.65 | 1.95 | 0.84 | 1.55 | 0.54 | 1.93 | 0.88 |
| Depressive symptoms (CES-D) | 17.82 | 7.42 | 19.43 | 8.58 | 15.20 | 8.08 | 17.25 | 7.84 | 14.36 | 8.26 | 16.01 | 8.62 | 10.06 | 6.92 | 12.96 | 7.41 |
| Quality of life (SF-12), physical health | 51.30 | 6.41 | 51.49 | 6.82 | 50.24 | 6.91 | 50.48 | 6.62 | 51.53 | 4.97 | 52.64 | 5.51 | 51.60 | 6.15 | 52.29 | 5.85 |
| Quality of life (SF-12), mental health | 34.03 | 9.47 | 35.61 | 10.48 | 40.63 | 8.92 | 39.76 | 10.55 | 42.37 | 9.14 | 40.57 | 11.47 | 44.54 | 8.86 | 39.94 | 11.82 |
