# Supplementary material for: Evaluating a Hybrid Web-Based Training Program for Panic Disorder and Agoraphobia: Randomized Controlled Trial
Source: J Med Internet Res. 2021 Mar 4;23(3):e20829. doi: 10.2196/20829 (PMC7974752; doi:10.2196/20829)
Supplement: Multimedia Appendix 3 [file jmir_v23i3e20829_app3.docx]

| Table 5. Differences between Groups at T2, T3 and T4 (intention-to-treat, N=92). | | | | | | | | | | | | | | | | |
| --- | --- | --- | --- | --- | --- | --- | --- | --- | --- | --- | --- | --- | --- | --- | --- | --- |
| Outcome | Between-groups effect T2 ^a^ | | | | | Between-groups effect T3 ^a^ | | | | | Between-groups effect T4 ^a^ | | | | | |
|  | Partial *η^2^* | Cohen’s *d* | 95% CI | ANCOVA ^b^ | | Partial *η^2^* | Cohen’s *d* | 95% CI | ANCOVA ^b^ | | Partial *η^2^* | Cohen’s *d* | 95% CI | ANCOVA ^b^ | |  |
|  |  |  |  | *F* ^c^ | *P*-value |  |  |  | *F* ^c^ | *P*-value |  |  |  | *F* ^c^ | *P*-value |  |
| Panic and agoraphobia severity, self-rating (PAS) | 0.10 | 0.66 | 0.24-1.08 | 9.77 | 0.002 | 0.16 | 0.89 | 0.46-1.31 | 17.40 | <0.001 | 0.14 | 0.81 | 0.38-1.24 | 14.63 | <0.001 |  |
| Anxiety symptoms, observer-rating (HAM-A) | 0.03 | 0.42 | 0.01-0.84 | 3.97 | 0.05 | NA | NA | NA | NA | NA | 0.05 | 0.47 | 0.05-0.88 | 4.86 | 0.03 |  |
| Agoraphobic cognitions (ACQ) | 0.06 | 0.51 | 0.05-0.93 | 5.88 | 0.02 | 0.07 | 0.55 | 0.14-0.97 | 6.80 | 0.01 | 0.05 | 0.46 | 0.04-0.87 | 4.69 | 0.03 |  |
| Body sensations (BSQ) | 0.05 | 0.46 | 0.05-0.88 | 4.77 | 0.03 | 0.14 | 0.79 | 0.37-1.22 | 13.96 | <0.001 | 0.09 | 0.66 | 0.22-1.06 | 9.01 | 0.003 |  |
| Agoraphobic avoidance (MI), accompanied | 0.00 | 0.06 | -0.35-0.47 | 0.12 | 0.73 | 0.01 | 0.24 | -0.17-0.65 | 1.23 | 0.27 | 0.03 | 0.32 | -0.09-0.73 | 2.31 | 0.13 |  |
| Agoraphobic avoidance (MI), alone | 0.05 | 0.45 | 0.04-0.86 | 4.49 | 0.04 | 0.03 | 0.36 | -0.05-0.78 | 2.90 | 0.09 | 0.11 | 0.70 | 0.27-1.12 | 10.72 | 0.002 |  |
| Depressive symptoms (CES-D) | 0.01 | 0.18 | -0.23-0.59 | 0.75 | 0.39 | 0.00 | 0.11 | -0.30-0.52 | 0.24 | 0.62 | 0.06 | 0.49 | 0.07-0.90 | 5.37 | 0.02 |  |
| Quality of life (SF-12), physical health | 0.00 | 0.0 | -0.41-0.41 | 0.01 | 0.91 | 0.02 | 0.28 | -0.13-0.69 | 1.72 | 0.19 | 0.00 | 0.13 | -0.28-0.54 | 0.33 | 0.57 |  |
| Quality of life (SF-12), mental health | 0.02 | 0.25 | -0.16-0.66 | 1.39 | 0.24 | 0.02 | 0.31 | -0.10-0.66 | 2.23 | 0.14 | 0.11 | 0.70 | 0.28-1.12 | 10.98 | 0.001 |  |
| ^a^ Missing data imputed by multiple imputation.  ^b^ Controlling for pre-treatment scores (T1).  ^c^ Degrees of freedom not provided due to multiple imputation. | | | | | | | | | | | | | | | |  |
